# Supplementary material for: Compliance to HIV testing and counseling guidelines at antenatal care clinics in the Kassena-Nankana districts of northern Ghana: A qualitative study
Source: PLoS One. 2022 Sep 30;17(9):e0274871. doi: 10.1371/journal.pone.0274871 (PMC9524674; doi:10.1371/journal.pone.0274871)
Supplement: S1 File — (DOCX) [file pone.0274871.s001.docx]

**Appendix II: Interview Guide for Health Workers**

**Compliance of HIV Testing and Counseling Study**

***[Start audio recording with permission of respondent]***

1. **Demographics**
2. What is your age?
3. What is your gender?
4. What is your highest level of education?
5. Have you graduated from midwifery school?
6. Have you been trained on HIV testing and counseling?
7. How many years have you worked as a midwife/public health nurse?
8. **Knowledge and Information**

The first part of the interview is about the kinds of information you provide during HIV testing and counseling during ANC visits.

1. Tell me about the process of HIV testing and counseling at ANC.
2. What information do you give to clients about HIV testing and counseling?

*[If respondent doesn’t mention any of the below topics, ask probing questions.]*

1. Do you provide pre-test counseling on HIV? If yes, what kinds of information do you provide?
2. What information do you provide about what HIV is?
3. What information do you provide about the risk factors of HIV?
4. What information do you provide about the modes of HIV transmission?
5. What information do you provide about how mother-to-child transmission occurs?

*[If respondent doesn’t mention during pregnancy, labor and delivery, and/or breastfeeding, ask if they provide information about these modes of transmission to their clients.]*

1. What kinds of information do you provide about the potential health problems associated with HIV that can occur during pregnancy?
2. What information do you provide about the purpose of HIV testing?
3. What information do you provide about the benefits of testing?
4. What information do you provide about the voluntary nature of HIV testing?
5. What information do you provide to your clients about their HIV test results being disclosed to other people?
6. What kinds of information do you provide on safe infant feeding practices in the case of HIV infection?
7. Do you provide post-test counseling on HIV? If yes, what kinds of information do you provide?
8. **Experience of HIV testing and counseling**

Now, I would like to ask you about your experience of HIV testing and counseling.

1. Do you offer HIV testing and counseling services to every client? Why or why not?
2. Do you inform your clients that they will be tested for HIV? Why or why not?
3. At what point during the pregnancy do you offer to be tested for HIV?
4. What happens if a woman initially declines an HIV test?
   1. If a woman initially declines an HIV test, are they routinely offered testing during subsequent visits?
   2. If a woman initially declines an HIV test, what kinds of information and counseling do you provide to them to encourage them to agree to be tested?
5. What happens after women are either identified as HIV-negative or HIV-positive?
   1. Do HIV-negative pregnant women receive counseling? If yes, what kinds of information do you provide?
   2. For those women who are found to be positive, do you give the appropriate antiretroviral treatment to the women?
   3. What kind of counseling and support do you provide to HIV-positive women?
   4. What kind of follow up care and treatment do you provide to HIV-positive women?
6. Should women who have HIV get the same care or different care to women who do not have HIV? Why or why not? If different, how should it be different?
7. How do you inform your clients of their test results?
8. Do you invite your clients’ partners for HIV testing and counseling? Why or why not?
9. What do you think about the importance of testing your clients’ partners for HIV?
10. What happens in the event that a woman has an unknown HIV status at the time of labor and delivery?
    1. Are women with unknown HIV status at the time of labor offered HIV testing and counseling?
11. What happens to women whose HIV status is unknown postpartum?
    1. Are women whose HIV status is unknown postpartum offered HIV testing and counseling?
    2. In the situation where the mother’s HIV status is unknown postpartum and she is unavailable to be tested and counseled, are newborns tested within 48 hours postpartum?
    3. If the test indicates that the baby is positive, what happens? Is the recommended antiretroviral prophylaxis offered?
12. What is done in the case of an indeterminate result?
13. What are the attitudes of pregnant women towards HIV testing and counseling?
14. What do you think your clients are doing to protect themselves and their children after receiving all this information?
15. How do you feel about your qualifications to provide HIV testing and counseling services?
16. What do you think about the time you spend explaining things to your clients?
17. What do you think about the information you provide to your clients?
18. How has your experience with involving your clients in making decisions about their antenatal care been?
19. What do you think about the quality of care you provide with regard to HIV testing and counseling?
20. **Perceptions of the HIV opt-out policy**

Now I would like to ask you several questions regarding your perception of the HIV opt-in and opt-out policies.

1. Can you describe what the HIV opt-out policy is?
2. What do you think about the HIV opt-out policy?
3. Do you think the current HIV opt-out policy is beneficial/useful? Why?
4. Do you think the current HIV opt-out policy is acceptable? Why?
5. What are some revisions to the current policy that could be made to improve it?
6. **Closing Question**
7. Is there anything else you would like to tell me about your thoughts and experiences with HIV testing and counseling?

Once again, thank you very much for taking the time to talk to me.

***[Stop Tape]***
