# Supplementary material for: Compliance to HIV testing and counseling guidelines at antenatal care clinics in the Kassena-Nankana districts of northern Ghana: A qualitative study
Source: PLoS One. 2022 Sep 30;17(9):e0274871. doi: 10.1371/journal.pone.0274871 (PMC9524674; doi:10.1371/journal.pone.0274871)
Supplement: S2 File — (DOCX) [file pone.0274871.s002.docx]

**Appendix I: Interview Guide for Mothers**

**Compliance of HIV Testing and Counseling Study**

***[Start audio recording with permission of respondent]***

**I.**               **Demographics**

First, I would like to ask you a few simple questions about yourself.

1. What is your age?
2. What is your gender?
3. What is your highest level of education?
4. How many children have you given birth to?

**II.**             **Knowledge and Information**

The first part of the interview is about the kinds of information you received during HIV testing and counseling during your ANC visits.

1. What usually happens during HIV testing and counseling at ANC?
2. What information is provided to you during HIV testing and counseling?

*[If respondent doesn’t mention any of the below topics, ask probing questions.]*

1. Did you receive pre-test counseling on HIV? If yes, what kinds of information did you receive?
2. What information did you receive about what HIV is?
3. What information did you receive about the risk factors of HIV?
4. What information did you receive about the modes of HIV transmission?
5. What information did you receive about how mother-to-child transmission of HIV occurs?

*[If respondent doesn’t mention that it can occur during pregnancy, labor and delivery, and/or breastfeeding, ask if they received information about these modes of transmission from their health provider.]*

1. What kinds of information did you receive about the potential health problems associated with HIV infection that can occur during pregnancy?
2. What information did you receive about the purpose of HIV testing during pregnancy?
3. What information did you receive about the benefits of HIV testing?
4. What information did you receive about the voluntary nature of HIV testing?
5. What information did you receive about your HIV test results being disclosed to other people?
6. Did you receive post-test counseling on HIV? If yes, what kinds of information did you receive?
7. What kinds of information did you receive on safe infant feeding practices in the case of HIV infection?

**III.**         **Experience of HIV testing and counseling**

Now I would like to ask you about your experience with HIV testing and counseling.

1. Were you tested for HIV during ANC?
   1. Were you informed that you would be tested for HIV?
   2. How did you feel when you were told that you would be tested for HIV?
   3. Did you have the freedom to choose if you wanted to be tested for HIV?
   4. At what points during your pregnancy were you tested for HIV?
   5. Has there been a time when you couldn’t be tested for HIV because they ran out of test kits?
2. What happens if you are not tested for HIV?
3. Did your health provider inform you of your test results? If yes, how were these results shared?
4. Was it done privately without the presence of other people or were there other people around?
5. Was it done in a private room or an open space?
6. Has there been a time when you were tested for HIV but were not sure of the results?
7. Did you share your results with your partners? If yes, how did you share your results with your partner?
8. Was your partner invited for HIV testing and counseling? Why?
9. What was his response when he was invited to be tested for HIV?
10. Did your partner go to get tested for HIV?
11. What do you think about your partner being tested for HIV?
12. Were any other family members invited for HIV testing?
13. What kind of follow-up care did you receive after delivery?
14. Did the midwife go to your home to visit you?
15. Have you ever attended a pregnancy school or durbar? If yes, what did they teach you there?
16. Has there ever been a time when the midwife brought an HIV-positive woman to show you as an example?
17. What do you think about how clearly the midwife explained the information to you?
18. What do you think about the time the midwife spent explaining things to you?
19. Was the time she spent on HIV counseling during the first ANC visit the same or different compared to the time she spent on HIV counseling during following visits?
20. What do you think about the way the midwife provided HIV testing and counseling services to you?
21. Do you think you can protect yourself and your child from HIV with the counseling you have received? Why?
22. How was your experience of being involved in making decisions about your antenatal care with respect to HIV testing and counseling?
23. How satisfied are you with the HIV testing and counseling you received? Why?

**IV.**            **Perceptions of the HIV opt-out policy**

Now I would like to ask you several questions regarding your perception of the HIV opt-out policy.

1. What do you think about the current way HIV testing and counseling is done?
2. Do you think HIV testing and counseling is beneficial/useful? Why?
3. Do you think HIV testing and counseling is acceptable? Why?
4. What are some problems and challenges with the way HIV testing and counseling is done?
5. What are some revisions to the current way HIV testing and counseling is done that could be made to improve it?

**V.**   **Closing Question**

1. Is there anything else you would like to tell me about your thoughts and experiences with HIV testing and counseling?

Once again, thank you very much for taking the time to talk to me.

***[Stop Tape]***
