# Supplementary material for: Compliance to HIV testing and counseling guidelines at antenatal care clinics in the Kassena-Nankana districts of northern Ghana: A qualitative study
Source: PLoS One. 2022 Sep 30;17(9):e0274871. doi: 10.1371/journal.pone.0274871 (PMC9524674; doi:10.1371/journal.pone.0274871)
Supplement: Attachment — Submitted filename: Response to Reviewers.docx [file pone.0274871.s003.docx]

**Manuscript Number: PONE-D-20-35332**

**Response to Reviewers**

**Comments from the Editor:**

1) Please ensure that your manuscript meets PLOS ONE's style requirements, including those for file naming. The PLOS ONE style templates can be found at

<https://journals.plos.org/plosone/s/file?id=wjVg/PLOSOne_formatting_sample_main_body.pdf> <https://journals.plos.org/plosone/s/file?id=ba62/PLOSOne_formatting_sample_title_authors_affiliations.pdf>

**Response: We have reviewed both PDFs detailing PLOS ONE’s style requirements and have edited the manuscript to reflect this.**

2) Please include additional information regarding the qualitative questionnaire used in the study and ensure that you have provided sufficient details that others could replicate the analyses. For instance, if you developed a questionnaire as part of this study and it is not under a copyright more restrictive than CC-BY, please include a copy, in both the original language and English, as Supporting Information, or include a citation if it has been published previously.

**Response: We have uploaded a copy of the qualitative interview guide for mothers and health workers in English as Supporting Information. During the data collection process, the research assistant (JA) who conducted interviews with the mothers in their preferred language used the English interview guides but verbally conveyed the questions in the local language. This research assistant was extensively trained by an experienced field interviewer to accurately translate the questions from English to the local languages, identifying key words in English that may not have a direct translation. This training included mock interviews in the local language with real-time feedback to ensure understandability and veracity of the translation.**

3) In the Methods, please discuss whether and how the questionnaire was validated and/or pre-tested. If these did not occur, please provide the rationale for not doing so.

**Response: This information is described in the original manuscript in the “Data Collection” paragraph in lines 177-180. The interview guides were pre-tested at the district hospital, War Memorial Hospital, in Navrongo with one mother and one midwife to assess the appropriateness of the questions and to determine the approximate time required to complete an interview. After this pre-test, we revised the interview guides based on the interviewers’ experiences and observations to improve the understandability of the questions.**

4) We note that you have indicated that data from this study are available upon request. PLOS only allows **data to be available upon request if there are legal or ethical restrictions on sharing data publicly. For** information on unacceptable data access restrictions, please see <http://journals.plos.org/plosone/s/data-availability#loc-unacceptable-data-access-restrictions>.

In your revised cover letter, please address the following prompts:

a) If there are ethical or legal restrictions on sharing a de-identified data set, please explain them in detail (e.g., data contain potentially identifying or sensitive patient information) and who has imposed them (e.g., an ethics committee). Please also provide contact information for a data access committee, ethics committee, or other institutional body to which data requests may be sent.

b) If there are no restrictions, please upload the minimal anonymized data set necessary to replicate your study findings as either Supporting Information files or to a stable, public repository and provide us with the relevant URLs, DOIs, or accession numbers. Please see http://www.bmj.com/content/340/bmj.c181.long for guidelines on how to de-identify and prepare clinical data for publication. For a list of acceptable repositories, please see http://journals.plos.org/plosone/s/data-availability#loc-recommended-repositories.

We will update your Data Availability statement on your behalf to reflect the information you provide.

**Response: The interview transcriptions cannot be shared publicly as it contains personally identifiable information and would therefore undermine the minimal risk ethical committee agreement and terms and conditions of consent. Interviews were confidential to enable freedom of expression, and participants consented to the study with the understanding that only anonymized quotations would be used in publications, not the entirety of the transcripts. Therefore, only illustrative excerpts from the transcripts, which qualify as the minimal data set, are included in the paper. Anonymized data can be made available to qualified researchers by request to the head of the data repository of the Navrongo Health Research Center: Peter Wontou (**[**peter.wontuo@navrongo-hrc.org**](mailto:peter.wontuo@navrongo-hrc.org)**).**

**Comments from Reviewer 1:**

1) Introduction section: Update literature on prevalence of HIV among pregnant women at ANC in study area. There is also relevant literature in Ghana e.g by Osei et al 2015 on prevention of MTCT of HIV and should be included. Probable reason for very low uptake of ARV services among HIV-positive pregnant women should be stated.

**Response: Updated data on the prevalence of HIV among pregnant women in the study area at the national (lines 65-67), regional (lines 80-82), and district level (lines 82-84) was included. Findings from the Osei et al. 2016 study on PMTCT of HIV is described in the fifth paragraph (lines 85-93) to highlight the rationale of our study. The probable reasons for low ARV coverage among HIV-positive pregnant women was added (lines 72-74).**

2) Study location: Provide more information on the health system context by way of facilities or services available at the different levels of health facilities especially as it relates to ANC and prevention of MTCT. Dates or period study was conducted should be stated.

**Response: The structure of Ghana’s health care system as organized by different levels of health facilities as described in lines 101-106 of the “Study Location” section. The following paragraph (lines 107-117) provides information on the HIV-related/PMTCT services that are available at the various levels of facilities. The dates of the data collection phase were added in lines 202-203 under the “Data Collection” section.**

3) Population and sampling approach: Consider systematically organizing this section into paragraphs of first the study population, sampling at the district level, zones and health facilities by levels of care for clarity. Please indicate the reasons for the sampling approaches used for the different populations.

**Response: As suggested by the reviewer, we have restructured the “Study Population and Sampling Approach” section for clarity. The “Study Population” section (lines 154-158) describes the study population. The “Sampling Approach” section (lines 159-174) was reorganized to funnel from district zones, health facilities, and participants. Thank you for this insightful suggestion.**

4) Results: Please provide demographic characteristics of study participants. Please include information on availability of treatment for the prevention of MTCT of HIV since it could also impact on compliance to the guidelines being assessed. Additionally it could be an important discussion point.

**Response: A table of demographic characteristics of study participants was added to the Results section in lines 235-236. Excerpts from the interviews describing the availability and accessibility of antiretrovirals for the treatment of HIV at ANC clinics were included in the “Process of HIV Testing and Counseling” of the Results section in lines 318-332.**

5) Consenting: Reason provided for oral consenting is not accurate since most respondents were health workers and may be considered as a limitation instead.

**Response: Thank you for pointing this out. We agree with this comment and have updated the statement about the reason for oral consent in the “Statement of Ethics” section (lines 230-231). We have amended the statement to read more appropriately.**
