# Supplementary material for: Compliance to HIV testing and counseling guidelines at antenatal care clinics in the Kassena-Nankana districts of northern Ghana: A qualitative study
Source: PLoS One. 2022 Sep 30;17(9):e0274871. doi: 10.1371/journal.pone.0274871 (PMC9524674; doi:10.1371/journal.pone.0274871)
Supplement: Attachment — Submitted filename: 2) Response to Reviewers.docx [file pone.0274871.s004.docx]

**Manuscript Number: PONE-D-20-35332**

**Response to Reviewers**

**Comments from Reviewer 1:**

First, I should say, I have enjoyed reading this piece of work. Understanding adherence to a given guideline in a clinical setting is an important area of research that potentially may inform the effort to enhance the quality of HIV testing services in Ghana in particular and SSA in general. I only have a few comments to enhance the manuscript's readability and technical soundness.

*Introduction*
Some of the paragraphs need a revisit. For example, the paragraph from 55-63 is hard to follow, given that it began with a very long sentence. It also specifies "The third and fourth components of this strategy . . .", but not sure if the reader will pick up which third & fourth components the authors are referring to.

**Response: We reformatted this sentence into a list for clarity (lines 56-62).**

It would be more informative in lines 65-67 if the authors provided the period and the change in ANC service use proportion.

**Response: We provided quantitative data from prior research to illustrate the increase in ANC use from 2006 to 2017-2018 (lines 68-69).**

*Methods*
The methods section provides a helpful and detailed description of how the study was conducted to address the research question. A few things to have a look at:

The authors have indicated in Line 149-150 that Grounded Theory underpinned the study design. There are various schools of thought on Grounded Theory as methodology and method one can apply in qualitative studies. Please provide more information on which (whether the classical/traditional or constructive grounded theory informed the inquiry process – subsequently reflect how the framework informed your process of data collection, sampling, & analysis. Please also include references in your method section as appropriate.

**Response: We used the Straussian grounded theory. We utilized the fundamental characteristics of this approach, namely theoretical sampling, thematic saturation, constant comparative analysis, and memoing to guide the sampling approach, data collection, and data analysis, which is further discussed in the Data Collection section (lines 247-253).**

In Line 160-163, the authors described as they have used theoretical sampling, which allows them to set criteria based on the research question, and ongoing analysis to recruit more participants from various population groups and settings. They have also said they used random sampling to document ethnic variation of any. This looks a purposive sampling as a random sample is not commonly used term in a qualitative study.

**Response: In the Kassena-Nankana districts, the Kassena ethnic group predominantly lives in the North and West zones while the Nankani ethnic group predominantly lives in the East and South zones. To ensure our study population included both ethnic groups, we randomly selected a zone to represent the Kassenas and a zone to represent the Nankanis. When selecting the health facilities, we used purposive sampling to select those that had a midwife providing antenatal care.**

As the authors have provided background to describe the health care system in Uganda, several health facilities provide ANC services with midwives. So, in Lines 144-165, it would help your readers know the types and how many health facilities were selected. Also, please explain why you selected those health facilities. This is crucial to the research objective provided in the paper, which aims to provide crucial information on whether there is adherence to the guide inline of HIV testing across different health facilities.

Response: We specified the number and types of health facilities in our sample (lines 203-204).

*Data analysis and Results*
The result section presented the findings using a pre-defined sub-heading/themes that are likely driven from the interview guides. As the authors have used open, axial, and selective coding underpinned by grounded theory, it would be more informative to present here the key themes that were selected/emerged from the account of participants than using themes of the interview guides. For instance, “Lack of privacy and breach of confidentiality” could be one example theme that the authors discussed but presented under the process of HIS Testing and counseling at ANC.

**Response: We organized the result section into the broader themes of the process of HTC, information provided during HIV counseling, voluntariness of HTC, and challenges with HTC to provide a general overview of the way in which HTC is provided. We agree that the theme of “Lack of Privacy and Breach of Confidentiality” is one that can stand alone, so we have made it a separate section (lines 532-544).**

In Line 215, it says the transcripts were corrected to facilitate coding. Not sure what the authors mean by that. Changing participants' wording while doing a verbatim transcription will risk the principle of authenticity, but there are situations where making the change are reasonable. It could be to maintain the grammatical flow of sentences as sometimes participants may provide an incomplete speech. Or to make an oral speech into written sentences and paragraphs. This has to be reflected in the quotes used in the result section. Some of the quotes are hard to follow. Line 326-330 is a good example of a quote with many pronouns, which makes it hard to follow the message. This is a typical example that transcribing correction/changing or adding additional information within [ ] required to help readers understand what the participant is referring to when they are using pronouns. Please revisit the quotes to ensure its readability.

**Response: The transcripts were edited to maintain grammatical flow and proper syntax of sentences as some of the interviews were conducted in the local languages and the transcribers had little time to review the grammar and syntax of their transcriptions. The edits were minimal, and care was taken to preserve the meaning of the participants’ responses. We have also edited the quotes to improve comprehension.**

Involvement of male partners in either using the services or women's decision making to use HIV testing services seems overlooked in the method (Line 128-146), result (line 334-335 & 340 – "male unfriendly perception among healthcare providers"), and discussion section (line 552). Please provide context if the guideline also addresses how male partners should be involved in PMTCT & how the services providers should engage with them.

**Response: The national guidelines on PMTCT indicate that HIV testing and counseling services should be encouraged and offered to male partners as well but does not provide further information on how service providers should engage with them. This has been incorporated in the Methods section (lines 179-180).**

There are a few grammatical issues (e.g., Line 280), and a few hard-to-follow sentences, especially the quotes (lines 343-344).

**Response: We have edited the grammar in Line 280 (now line 314) and the quote in Lines 343-344 (now lines 413-414) to improve readability.**

*Discussion*
If key themes emerged/were selected from the open, axial, and selective coding process, readers would expect a summary paragraph at the beginning of the discussion section. Since the results are presented differently, the discussion started with one of the key themes – setting HTC as a compulsory vs. voluntary option. Please reconsider revising the paragraph to summarize the attributes/themes that show loose adherence to the national guidelines on HTC in ANC settings.

**Response: We revised the first paragraph of the discussion to summarize the attributes/themes that show loose adherence to the national guidelines on HIV testing and counseling in antenatal care settings (lines 622-629).**

*Limitation*
The study to assess whether healthcare provides comply with the national guidelines on HIV testing used snowball sampling (Line 172-174) of women who used the services may have created bias. There is a chance that the healthcare providers are likely to suggest recruiting women whom they think can speak the best experiences they had in the health facilities when they receive HIV testing. As such, this limitation has to be reflected in the limitation of the study. Also, the study used interviews to assess compliance of a practice. A study design like ethnography would be more stronger to provide the existing practice of HIV testing. This can be reflected in the limitation as well.

**Response: We agree with the reviewer’s additional suggested limitations and have edited the limitation section to reflect this (lines 717-730).**

*Conclusion*
In Line 604-615, strong recommendations have been made. However, based on this single study, the reader may not know if there are ongoing efforts related to the actions listed within the recommendations made by the authors. It is also not clear whether these actions recommended by the authors can be taken as effective measures to address the grab identified regarding healthcare providers' compliance in providing HIV testing services. Instead, it would be more pragmatic to suggest that more studies be conducted using different research designs (such as ethnography or observational quantitative designs) to directly measure healthcare providers' compliance in providing HIV testing and counseling services against the national guideline.

**Response: The recommendations were made to offer potential interventions and strategies to improve standardization and quality of HIV testing and counseling services. We agree with the reviewer that further research, such as ethnographies and observational studies, should be conducted to capture actual practices of HIV testing and counseling and have revised the conclusion to reflect this (lines 757-759).**
